# The Use of Parsimonious Questionnaires in Occupational Health Surveillance: Psychometric Properties of the Short Italian Version of the Effort/Reward Imbalance Questionnaire

**DOI:** 10.1100/2012/372852

**Published:** 2012-08-13

**Authors:** Nicola Magnavita, Sergio Garbarino, Johannes Siegrist

**Affiliations:** ^1^Institute of Occupational Medicine, The Catholic University of the Sacred Heart, 00168 Rome, Italy; ^2^State Police Health Service Department, Ministry of the Interior, Italy; ^3^Department of Neuroscience, Ophthalmology and Genetics, University of Genoa, 16132 Genoa, Italy; ^4^Department of Medical Sociology, University of Duesseldorf, 40225 Duesseldorf, Germany

## Abstract

*Purpose*. To perform a parsimonious measurement of workplace psychosocial stress in routine occupational health surveillance, this study tests the psychometric properties of a short version of the original Italian effort-reward imbalance (ERI) questionnaire. *Methods*. 1,803 employees (63 percent women) from 19 service companies in the Italian region of Latium participated in a cross-sectional survey containing the short version of the ERI questionnaire (16 items) and questions related to self-reported health, musculoskeletal complaints and job satisfaction. Exploratory factor analysis, internal consistency of scales and criterion validity were utilized. *Results*. The internal consistency of scales was satisfactory. Principal component analysis enabled to identify the model's main factors. Significant associations with health and job satisfaction in the majority of cases support the notion of criterion validity. A high score on the effort-reward ratio was associated with an elevated odds ratio (OR = 2.71; 95% CI 1.86–3.95) of musculoskeletal complaints in the upper arm. *Conclusions*. The short form of the Italian ERI questionnaire provides a psychometrically useful tool for routine occupational health surveillance, although further validation is recommended.

## 1. Background

Work-related stress is one of the leading causes of workers' ill health in developed countries [1], where it has considerable effects on sickness absence and disability [2]. Epidemiological investigations document significant associations of stressful work with coronary heart disease [3–5], depression [6–8], musculoskeletal disorders [9, 10], and other stress-related health problems [11]. In recent years, specific theoretical concepts and their measurement in terms of psychometrically validated standardized questionnaires has led to progress in identifying psychosocial working conditions that produce adverse health effects.

Occupational epidemiological research has focused on two such concepts: the “demand-control” model and the “effort-reward imbalance” model. The former assumes that job task profiles characterized by high psychological demands and a low level of control and decision latitude are stressful [12, 13], whereas the latter is concerned with stressful features of the work contract resulting from failed reciprocity between (high) efforts spent and (low) rewards received [14, 15]. In this model, three types of rewards are important: money, esteem, and career, including job security. Moreover, in addition to extrinsic effort this model assesses a distinct personal pattern of coping with job demands, termed overcommitment. According to this theoretical approach, workers who experience high effort, low reward, and a mismatch between them (high cost-low gain), and workers who exhibit a high level of overcommitment are susceptible to an elevated risk of stress-related disorders.

The two models complement each other, and some evidence indicates that the former concept is of particular use in industrial workers, whereas the latter may be more appropriate in the tertiary sector [16, 17]. In several countries, including Italy, routine assessment of psychosocial stress at work has become compulsory for occupational health services. Therefore, short, validated, and easily applicable questionnaires are needed to assess the prevalence of work-related stress as a basis for potential preventive efforts. A few years ago, a short version of the original questionnaire used to measure the effort-reward imbalance (ERI) model [14, 15] was developed and tested in a German [18] and a Swedish [19] sample of male and female workers. We set out to conduct a psychometric test of the short version of the original Italian questionnaire [20] in a large sample of male and female employees in Italy. More specifically we aim at replicating its construct validity and analyzing its association with job satisfaction, musculoskeletal complaints, and self-rated health.

## 2. Methods

In 2010, workers undergoing regular health surveillance in the workplace under the responsibility of the first author were asked to complete an anonymous questionnaire composed of three sections; (1) basic socio-demographic information (restricted to gender and age to ensure anonymity); (2) musculoskeletal disorders, general health and job satisfaction; (3) the short version of the ERI questionnaire. As mentioned, health surveillance is mandatory in Italy for workers exposed to occupational hazards, yet assessment procedures are flexible, thus, allowing some innovation—in our case the administration of the short ERI questionnaire. Workers who had been employed for at least one year in the same workplace were eligible (see sample description below). The study was approved by the Ethics Committee of the Catholic University of Sacred Heart.

Musculoskeletal complaints were assessed by the Nordic questionnaire [21]. Participants were asked to assess (1) if they had had problems (pain, ache, discomfort) at any time during the previous 12 months (recurrent symptoms); (2) if they had had problems during the previous 7 days (persistent symptoms); (3) if they were experiencing problems at the time of the medical examination (active symptoms). Those who gave a positive response were requested to specify in which of 9 parts of the body (neck, shoulders, elbows, wrist and hands, upper back, low back, hips and thighs, knees, ankles, and feet) they had experienced health problems. All items are binary, with the category “yes” defining presence and “no” defining absence of musculoskeletal symptoms. The questionnaire provides information about one-year prevalence and the point prevalence of musculoskeletal complaints.

General self-rated health was measured by the one-item question: “How would you rate your general state of health?”. Respondents answered on a scale from 1 to 5 ranging from 1 “I've serious health problems” to 5 “I've very good health.”

Job satisfaction was measured by a single item of the scale proposed by Warr et al. [22]: “How do you feel about your job as a whole?”. Respondent answered on a scale ranging from 1 “I'm extremely dissatisfied” to 7 “I'm extremely satisfied.”

Effort, reward, and overcommitment were measured by the short version of the ERI questionnaire where effort is measured by three questions, reward by seven questions, and overcommitment by six questions [18]. All respective items were answered on a four-point Likert scale (ranging from 1 “strongly agree” to 4 “strongly disagree”). Total scores of these ratings were calculated with appropriate recoding, so that high scores reflect high effort, high reward and high overcommitment. Thus, the range of effort scale is 3–12, of the reward scale is 7–28, and of the overcommitment scale is 6–24. Additionally, an effort-reward ratio was calculated by dividing the score of “effort” by the score of reward, adjusted for unequal number of items. This was done in order to quantify the degree of mismatch between effort and reward at individual level.

### 2.1. Sample

This voluntary survey was offered to all male and female workers from 19 different service companies who had been employed there for more than one year. Overall, 1,803 subjects (669 male, 37%, 1,134 female workers, 63%) completed the questionnaire and were included in the analysis. The response rate ranged from 89% to 100% (average: 95.3%). Given the high response rate there was no indication of selection bias. Workers were employed in health care services (*n* = 998, 55.4%), social services (*n* = 395, 21.9%), retail (*n* = 281, 15.6%), and financial services (*n* = 129, 7.1%) (Table 1).

### 2.2. Statistical Analysis

An exploratory factor analysis, with principal component extraction and varimax rotation method with Kaiser normalization was used to test the psychometric characteristics of the scales of the ERI short questionnaire. To identify the scales, we adopted the criteria that item loading should be greater than .50 on the loading factor and at least .20 above that for any other factors, thus, indicating that the item was conceptually distinct [23]. The resulting factor structure was compared to the original construct of the German version. Reliability of each subscale was calculated with the Cronbach's alpha statistic.

We tested some aspects of criterion validity of the short ERI version assuming that employees who scored high on the scales of the construct were at elevated risk of experiencing poor self-rated health and poor job satisfaction, compared to those with lower scores. The associations between exposure variables and outcomes were examined by multiple linear regression analysis. After first calculating an unadjusted model, we subsequently adjusted for age and gender, entering effort, reward and overcommitment as independent variables. We then repeated the same steps entering effort-reward ratio as an independent variable.

Finally, the association between exposure variables and musculoskeletal complaints was investigated by logistic regression analysis. First we calculated an adjusted model, and then we adjusted it for age and gender. Odds ratios and confidence intervals at 95% were calculated. We used version 15.0 of SPSS for Windows for the statistical analyses.

## 3. Results

### 3.1. Psychometric Properties

The factor analysis identified four factors that accounted for 53% of the total variance. After rotation, each factor was seen to correspond to a specific construct, as in the original version (Table 2). The first factor corresponded to the “overcommitment” scale, the second one to the “effort” scale, and the last two factors to the “reward” construct. The subdivision of “reward” into two factors, although already observed in a previous Italian study based on the original 23-item questionnaire [20], did not conform to the theoretical assumption of three subcomponents. Although “job security” was well replicated, there was no clear distinction between “esteem” and “salary, career prospects.” It seems that the items measuring non-monetary rewards (esteem) and those measuring monetary and status-related rewards “status” did not cluster in two separate factors. Almost all items showed significant factor loadings (>0.50) on the factor corresponding to their construct, and negligible loading (<0.20) on other factors; only two items pertaining to the “overcommitment” scale (oc1 and oc4) had loadings inferior to .50 on their factor, but their loadings on other scales were negligible, thus, providing evidence of their specificity.

Using factor analysis, we composed 4 scales (“effort,” “job security,” “esteem and status”, “overcommitment”) and calculated their internal consistency. Cronbach's alpha coefficients were 0.72 for “overcommitment”, 0.69 for “effort”, 0.68 for “esteem and status”, and 0.63 for “job security”, suggesting satisfactory internal consistency in view of the small number of items (Table 2). Item-total correlations coefficients (corrected) varied between 0.34 and 0.61 and were all above the threshold of 0.30 [24], indicating considerable consistency between items defining respective scales. Using the theoretical model, we constructed a general “reward” scale based on the two subscales, and a ratio of “effort” and “reward” was defined according to established procedures [15]. Thus, in further analyses that tested associations with health and job satisfaction, four explanatory variables were included (“effort”, “reward”, “effort-reward ratio”, “overcommitment”).

### 3.2. Associations with Health and Job Satisfaction

Multiple regression analyses showed that the “effort”, “reward” and “overcommitment” scales' were significantly related to self-rated general health. “Effort” had an inverse relationship, while “reward” and also “overcommitment” were positively related to the level of self-rated health. Correction for age and gender weakened the association between “effort” and health to some extent, while the relationships between “reward”, “overcommitment” and health remained unchanged. Low levels of “effort” and high levels of “reward” were significantly associated with job satisfaction, whereas there was no evident association with “overcommitment”. Significant relationships were observed between the “effort-reward ratio” and self-rated health as well as job satisfaction. As illustrated in Table 3, scoring low on the “effort-reward ratio” was associated with good self-rated health and high job satisfaction (Table 3).

Logistic regression analysis revealed that scoring high on “reward” decreased the probability of reporting low back pain at the time of the medical examination (OR = 0.87, 95% CI 0.84–0.90), while “overcommitment” was associated with an elevated odds ratio (OR = 1.16, 95% CI 1.11–1.20). “Reward” (OR = 0.81, 95% CI 0.78–0.84) and “overcommitment” (OR = 1.18, 95% CI 1.12–1.23) were associated in the same way with upper limb disorders. Additional adjusting for age and gender did not substantially change the results (Table 4). Finally, the “effort-reward ratio” was significantly related to upper limb disorders (OR = 3.37, 95% CI 2.34–4.39), and this association remained significant after control of confounding factors (OR = 2.71, 95% CI 1.86–3.95).

## 4. Discussion

This study was set up to test the psychometric properties of the short version of the original questionnaire that measured occupational effort-reward imbalance in a convenience sample of Italian workers. Data show satisfactory internal consistency of these shortened scales, and exploratory factor analysis supported the conceptual distinction between the “effort” and “over commitment” scales. Contrary to most other studies [15, 18, 19, 25], but in keeping with a previous Italian investigation based on the original questionnaire [20], we observed two (instead of three) subcomponents of the construct “reward” as there was no clear distinction between nonmaterial and material components of job-related rewards. Although this result calls for further investigation, it may well be that it reflects the specific sample composition of this study which was largely composed of women working in health and social services where the material and nonmaterial rewards of professional work are often not separated as clearly as is the case in other branches and sectors of the labor market.

Model fits derived from confirmatory factor analysis were not tested, but in keeping with previous studies, a summary “reward” scale and a ratio quantifying the mismatch between effort and reward at individual level were constructed for further analyses of criterion validity. With this aim in mind, we analyzed associations with some health indicators (self-rated health and two types of musculoskeletal complaints). While results in general support the hypothesis, there were some noticeable exceptions.

First, “overcommitment” was positively associated with self-rated health and musculoskeletal complaints. This contradicts previous findings demonstrating reduced rather than enhanced self-reported health among overcommitted workers [18, 25, 26]. In the Italian sample, mean scores of “overcommitment” were not very high, and this fact may prevent the detection of adverse health effects. Secondly, while all associations of “reward” with the criterion variables are significant, there is an exception to this trend as regards the “effort-reward ratio” (low back pain). On the other hand, in accordance with the theoretical assumption, associations with the ratio are generally stronger than those observed with single scales of the model.

There are obvious limitations to this study. Firstly, data are drawn from a limited sample of tertiary sector companies, located in the Latium region of Italy and under the responsibility of a single physician. It is, therefore, not clear to what extent findings can be extended to other geographic areas or economic sectors in Italy. In this study, no measure of negative affectivity was available, so reporting bias cannot be excluded. We cannot exclude bias due to common method variance, given the fact that both work-related and health-related questions are based on subjective evaluations reported within the same assessment. The cross-sectional study design, with restriction to a single exposure assessment, precludes any reference to temporal or causal directions of observed statistical associations. Finally, we provide only overall results, without specifying them according to occupational categories, hazard exposure, socioeconomic conditions, education, or type and duration of employment.

Yet, this study confirms the usefulness of a newly developed short version of the ERI questionnaire in the Italian context, as its basic findings are in agreement with the results of two previously published reports from Germany and Sweden. Moreover, it is one of the first studies applying a 4-point Likert scale format for answering the items instead of the previous 5-point Likert scale which has been the subject of methodological criticism [27]. Finally, given the exceptionally high response rate of this survey, selection bias is virtually ruled out.

In conclusion, by balancing the limitations and strengths of this study, our results indicate that the short form of ERI provides a psychometrically useful tool for epidemiological studies at the workplace. Given the model's success in explaining work-related health risks it may be particularly useful for application in routine medical surveillance where parsimonious assessment has high priority.

## Figures and Tables

**Table 1:** Companies participating in the study and participation rates.

Name	Sector	*N* cases	% of sample	Eligible workers, *N *	Participation rate, %
A	Financial	34	1.9	35	97.1
B	Social services	42	2.3	44	95.5
C	Health care	158	8.8	163	96.9
D	Retail	24	1.3	26	92.3
E	Retail	53	2.9	55	96.4
F	Social services	206	11.4	208	99.0
G	Social services	39	2.2	43	90.7
H	Health care	193	10.7	202	95.5
I	Retail	38	2.1	40	95.0
L	Financial	28	1.6	29	96.6
M	Social services	88	4.9	91	96.7
N	Social services	20	1.1	22	90.9
O	Health care	79	4.4	82	96.3
P	Health care	268	14.9	285	94.0
Q	Health care	16	.9	18	88.9
R	Health care	284	15.8	312	91.0
S	Retail	142	7.9	144	98.6
T	Financial	67	3.7	68	98.5
U	Retail	24	1.3	24	100.0
V	Total	1803	100.0	1891	95.3

**Table 2:** Principal component analysis of ERI items with varimax rotation of factor loadings. The significant item loadings are highlighted by Bold.

Item no.	Mean (SD)	Components				Item-total correlation coefficient (corrected)
		1	2	3	4	
eri1 (§)—I have constant time pressure due to a heavy work load	2.46 (.79)	−.149	**.753**	−.140	−.081	.547
eri2 (§)—I have many interruptions and disturbances while performing my job	2.64 (.82)	−.057	**.727**	−.139	−.071	.486
eri3 (§)—Over the past few years, my job has become more and more demanding	2.14 (.76)	−.098	**.739**	−.003	−.015	.498
eri4 (§)—I receive the respect I deserve from my superior or a respective relevant person	3.17 (.72)	.082	−.051	**.702**	−.024	.402
eri5—My job promotion perspectives are poor	2.48 (.89)	−.022	−.219	.173	**.667**	.408
eri6—I have experienced or I expect to experience an undesirable change in my work situation	2.72 (.76)	.108	−.170	.234	**.717**	.518
eri7—My job security is poor	2.68 (.81)	.077	.119	.071	**.787**	.396
eri8 (§)—Considering all my efforts and achievements, I receive the respect and prestige I deserve at work	2.79 (.72)	−.016	−.051	**.791**	.102	.540
eri9 (§)—Considering all my efforts and achievements, my job promotion prospects are adequate	2.62 (.71)	.030	−.027	**.698**	.306	.540
eri10 (§)—Considering all my efforts and achievements, my salary/income is adequate	2.19 (.77)	−.128	−.120	**.546**	.226	.385
oc1—I get easily overwhelmed by time pressure at work	2.65 (.71)	**.305**	−.556	.006	.091	.350
oc2—As soon as I get up in the morning I start thinking about work problems	2.76 (.83)	**.686**	−.233	−.034	.101	.539
oc3 (§)—When I get home, I can easily relax and “switch off” work	2.79 (.77)	**.647**	.069	.268	−.045	.360
oc4—People close to me say I sacrifice too much for my job	2.46 (.74)	**.417**	−.295	−.104	.068	.344
oc5—Work rarely lets me go, it is still on my mind when I go to bed	2.85 (.76)	**.791**	−.168	−.022	.016	.613
oc6—If I postpone something that I was supposed to do today I'll have trouble sleeping at night	2.64 (.81)	**.713**	−.104	−.078	.043	.506

Construct		Overcommitment	Effort	Reward-respect	Reward-career	

% of variance explained by the scale		14.8%	14.1%	13.3%	11.1%	

Reliability (Cronbach's alpha)		.72	.69	.68	.63	

(§) reversed score.

**Table 3:** Associations of the scales of the short effort-reward imbalance questionnaire with self-rated general health and job satisfaction (multiple regression analyses).

ERI component	Self-rated general health			Job satisfaction		
		Corrected for age B			Corrected for age B	
	Uncorrected B (*P*)	(*P*)		Uncorrected B (*P*)	(*P*)	
		Male	Female		Male	Female
Effort	−0.128 (*P* < 0.001)	−0.07 (*P* < 0.05)	−0.064 (*P* < 0.05)	−0.105 (*P* < 0.001)	−0.141 (*P* < 0.001)	−0.080 (*P* < 0.01)
Reward	0.192 (*P* < 0.001)	0.198 (*P* < 0.001)	0.142 (*P* < 0.001)	0.368 (*P* < 0.001)	0.383 (*P* < 0.001)	0.355 (*P* < 0.001)
Overcommitment	0.119 (*P* < 0.001)	0.119 (*P* < 0.002)	0.095 (*P* < 0.001)	0.038 (n.s.)	0.039 (n.s.)	0.034 (n.s.)
Effort/Reward ratio	−0.273 (*P* < 0.001)	−0.256 (*P* < 0.001)	−0.166 (*P* < 0.001)	−0.390 (*P* < 0.001)	−0.427 (*P* < 0.001)	−0.365 (*P* < 0.001)

**Table 4:** Associations of the scales of the short effort-reward imbalance questionnaire with acute low back pain and musculoskeletal problems at the upper arm (logistic regression analyses) (Odds ratios and 95% confidence intervals (CI)).

	Unadjusted		Adjusted for age and gender	
	Odds ratio	95% CI	Odds ratio	95% CI
Low back pain				
Effort	0.94	0.88–1.00	0.94	0.88–1.00
Reward	0.87^∗∗∗^	0.84–0.90	0.88^∗∗∗^	0.85–0.91
Overcommitment	1.16^∗∗∗^	1.11–1.20	1.16^∗∗∗^	1.11–1.20
Effort/Reward ratio	1.10	0.83–1.45	1.15	0.88–1.50

Upper arm complaints				
Effort	1.10^∗^	1.02–1.18	1.06	0.98–1.14
Reward	0.81^∗∗∗^	0.78–0.84	0.82^∗∗∗^	0.78–0.85
Overcommitment	1.18^∗∗∗^	1.12–1.23	1.20^∗∗∗^	1.14–1.25
Effort/Reward ratio	3.37^∗∗∗^	2.34–4.39	2.71^∗∗∗^	1.86–3.95

^∗^Significant at *P* < 0.05; ^∗∗^significant at *P* < 0.01; ^∗∗∗^significant at *P* < 0.001.
